# Successful use of extracorporeal membrane oxygenation for life‐threatening macrophage activation syndrome after treatment with tocilizumab in an systemic juvenile idiopathic arthritis patient

**DOI:** 10.1002/pdi3.32

**Published:** 2023-10-06

**Authors:** Xi Yang, Yingfu Chen, Rongxin Dai, Yunfei An, Xin Yan, Xiaodong Zhao, Xuemei Tang

**Affiliations:** ^1^ Division of Rheumatology and Immunology Children's Hospital of Chongqing Medical University Chongqing China; ^2^ Ministry of Education Key Laboratory of Child Development and Disorders Children's Hospital of Chongqing Medical University Chongqing China; ^3^ Chongqing Key Laboratory of Child Infection and Immunity Children's Hospital of Chongqing Medical University Chongqing China; ^4^ Department of Critical Care Medicine Children's Hospital of Chongqing Medical University Chongqing China

**Keywords:** extracorporeal membrane oxygenation, macrophage activation syndrome, systemic juvenile idiopathic arthritis, tocilizumab

## Abstract

Macrophage activation syndrome (MAS) is a rare, potentially life‐threatening condition in rheumatic diseases. The primary treatments consist of high‐dose corticosteroids and immunosuppressive drugs, although more recently, cytokine inhibitors like anakinra or tocilizumab (TCZ) have been reported. We present a case of a child with systemic juvenile idiopathic arthritis (sJIA). After receiving a single infusion of TCZ, the child developed progressive hypoxia and was subsequently transferred to the pediatric intensive care unit (PICU) after 4 days. An immediate postintubation chest X‐ray revealed a diffuse exudative lesion. Despite continuous efforts to provide mechanical ventilation and respiratory support, the patient's oxygen saturation continued to decline. Moreover, the patient developed hemodynamic compromise, necessitating the administration of norepinephrine. Eventually, vasopressin and dopamine were added to maintain stable hemodynamics. After an intensive but ineffective treatment, extracorporeal membrane oxygenation (ECMO) was initiated in the PICU after 16 h. The patient successfully recovered and was weaned off ECMO support after 60 h. Following discharge from the PICU, given the severe refractory clinical features, we made an attempt to readminister TCZ treatment. However, within half an hour of TCZ infusion, the patient experienced anaphylaxis characterized by palpitations and chest tightness, leading to the discontinuation of TCZ. TCZ, as a biological agent, is commonly used in the treatment of sJIA. Nonetheless, the occurrence of MAS and anaphylaxis following TCZ administration for sJIA may be more prevalent than previously recognized. Pediatric rheumatologists should exercise caution when initiating TCZ for active sJIA. Furthermore, we want to underscore the importance of life‐saving techniques such as ECMO for sJIA patients in emergency situations.

## 1 BACKGROUND

Macrophage activation syndrome (MAS) is a life‐threatening complication of rheumatic diseases and refers to a condition caused by excessive activation and expansion of T lymphocytes and macrophagic histiocytes that exhibit hemophagocytic activity. It is most commonly observed in systemic juvenile idiopathic arthritis (sJIA). Early diagnosis is crucial as appropriate therapy can significantly improve outcomes. Tocilizumab (TCZ), an anti‐IL‐6 receptor antibody, inhibits IL‐6 signal transduction by selectively and competitively binding to the IL‐6 binding site of soluble and membrane‐bound IL‐6 receptors. TCZ has shown effectiveness in the treatment of sJIA. However, several recent reports have provided evidence that TCZ treatment is associated with MAS in sJIA.^1^, ^2^ Acute pulmonary edema with refractory shock is a rare and potentially fatal complication of MAS. Given the rarity of this condition, experience with its treatment is very limited. Venoarterial extracorporeal membrane oxygenation (VA‐ECMO) is increasingly being utilized to support severe pulmonary and/or cardiac failure. This paper presents our successful experience in treating acute pulmonary edema and refractory shock with ECMO following TCZ infusion.

## 2 PATIENT PRESENTATION

**FIGURE 1:**
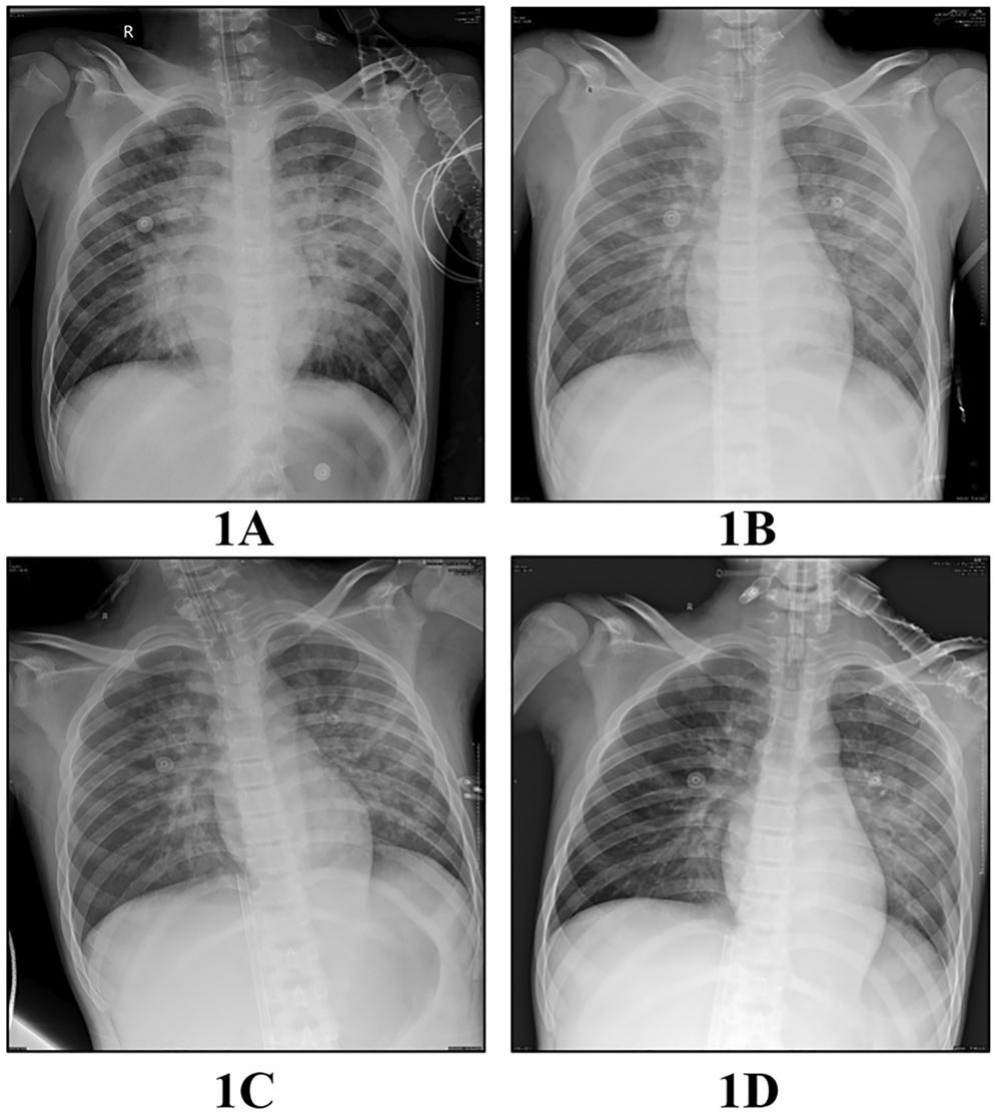
Chest X‐Ray during PICU admission. (A) Severe exudative lesions in both lungs after conventional mechanical ventilation (day 12); (B) Severe exudative lesions in both lungs after VA‐ECMO placement (day 13); (C) Improved exudative lesions in both lungs after VA‐ECMO placement (day 14); and (D) Resolution of most lesions in both lungs before PICU discharge (day 18). PICU, pediatric intensive care unit; VA‐ECMO, venoarterial extracorporeal membrane oxygenation.

**FIGURE 2:**
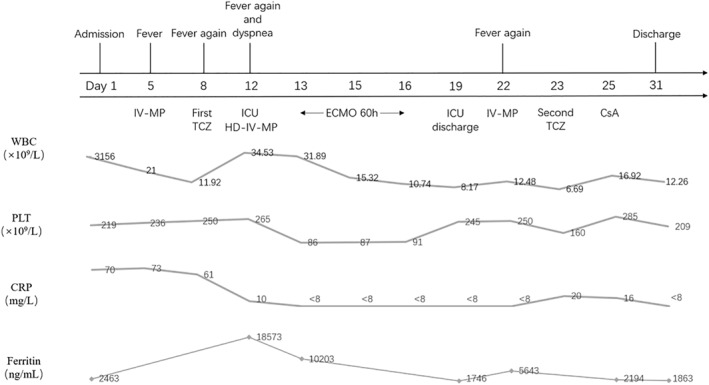
Correlation between clinical aspects, laboratory tests, and treatment of sJIA‐related MAS. CRP, C‐reactive protein; CSA, cyclosporine‐A; ECMO, extracorporeal membrane oxygenation; HD‐IV‐MP, high dose intravenous methylprednisolone; MAS, macrophage activation syndrome; PLT, platelets; sJIA, systemic juvenile idiopathic arthritis; TCZ, tocilizumab; WBC, white blood cells.

The patient, a previously healthy 14‐year‐old Chinese boy born to nonconsanguineous parents, presented with intermittent low‐grade fever and a skin rash that had been persisting for 2 weeks. Subsequently, he developed continuous high fever and general discomfort. His parents were healthy, and there was no family history of rheumatic or hematological diseases. Physical examination revealed scattered, slightly raised round erythematous lesions and shoulder joint pain. There was no evidence of splenomegaly or hepatomegaly. Laboratory tests showed a white blood cell count of 22.9 × 10^9^/L, hemoglobin level of 13.4 g/dL, platelet count of 302 × 10^9^/L, C‐reactive protein (CRP) level of 20 mg/L, erythrocyte sedimentation rate of 32 mm/1 h, ferritin level of 3289 ng/mL, fibrinogen level of 3.8 g/L, and triglyceride level of 1.5 mmol/L. Aspartate aminotransferase (AST), alanine amiotransferase, echocardiography, and bone marrow aspiration appeared to be normal. Serologic tests for Epstein–Barr virus, cytomegalovirus, human immunodeficiency virus, viral hepatitis, and Group A streptococcal infections were negative, as were serial blood and urine cultures. Based on the presence of fever, arthritis, and skin rash, sJIA was diagnosed according to international league of associations for rheumatology criteria.^3^ Treatment with intravenous methylprednisolone (2 mg/kg/day), ibuprofen, and methotrexate initially resulted in improvements in clinical and laboratory parameters. However, when the methylprednisolone dosage was tapered, the patient experienced a relapse of spiking fever, rash, and arthralgia. To enhance disease control and facilitate glucocorticoid withdrawal, we initiated TCZ treatment (8 mg/kg), which led to complete improvement in clinical and laboratory parameters. After 4 days of TCZ treatment with oral prednisolone (1 mg/kg/day), the patient developed a fever and acute dyspnea. He was urgently admitted to the Pediatric Intensive Care Unit (PICU), where inotropic and vasoactive support were optimized, and conventional mechanical ventilation was initiated shortly afterward. Physical examination revealed coarse breath sounds, and bilateral wet rales were observed in the lungs. The chest X‐ray displayed a diffuse exudative lesion (Figure 1A). Echocardiography demonstrated a slightly enlarged heart with decreased diastolic function but normal myocardial enzymes. Platelet counts decreased from 265 × 10^9^/L to 87 × 10^9^/L within 2 days, and serum ferritin levels increased to 18,573 ng/mL. AST levels rose to 216 IU/L, and fibrinogen levels dropped to 0.89 g/L. The patient's laboratory data fulfilled the PRINTO diagnostic criteria for MAS 2016.^4^ The patient initially received pulse methylprednisolone therapy, followed by a maintenance dosage of 10 mg/kg/day. Despite the administration of various drugs, including cardiotonics and vasopressors like norepinephrine, dopamine, and milrinone, the patient's blood pressure dropped to as low as 70/42 mmHg and could not be stabilized. The chest X‐ray still indicated the presence of a diffuse exudative lesion (Figure 1B). The patient's PF value is below 80, the blood vessel activity index is above 80, and they still have low blood pressure with lactate levels higher than 5. Unfortunately, even after 12 h of life support, normal blood pressure could not be maintained, leading to the initiation of VA‐ECMO. Specifically, a larger tube (19F) is put in the vein (MEDRONI CB96670‐019), and a slightly smaller tube (15F) is placed in the artery (MEDRONI CB96570‐015). At the same time, a smaller tube (6F) is used to provide additional blood flow through the leg. The ECMO circuit was composed of a Medos Hilite7000LT oxygenator with coated tubing (Sorin AB2414CN) and a Sorin pump (Revolution 5). The anticoagulation plan during ECMO involved intravenous heparin administration to maintain the activated partial thromboplastin time between 50 and 80 s and a whole blood Activated Clotting Time (ACT) of 180–220 s. Initially, the ECMO pump was set to flow at 3 L/min with a sweep gas of 3 L/min of oxygen (FiO_2_ of 100%). Immediate arterial blood gas studies conducted right after ECMO implementation demonstrated a swift resolution of hypoxia and improvement in acid–base balance. After 36 h, blood pressure was successfully maintained with fewer vasoactive drugs, and multiple follow‐up chest radiographs were performed (Figure 1C). Gradually, pulmonary edema improved, allowing for a gradual reduction of ECMO support. On Day 2.5 (60 h), the patient was definitively weaned off ECMO. Clinical testing showed improvements in MAS markers and the chest X‐ray (Figure 1D), and the patient was subsequently discharged from the PICU. To enhance disease control and facilitate glucocorticoid withdrawal, TCZ was administered again 14 days after the first infusion. However, 30 min after the second TCZ infusion, the patient experienced acute anaphylaxis, characterized by palpitations and chest tightness. The TCZ infusion was immediately stopped, and the patient quickly recovered with normalized MAS markers. With the full agreement of the patient's family, cyclosporine A was added to the treatment regimen. As the prednisolone dosage was gradually reduced, the patient showed improvement and was discharged from the hospital after 1 month. The relationship between clinical aspects, laboratory tests, and the treatment of sJIA‐related MAS is summarized in Figure 2. Currently, the patient has been in clinical remission under medication for nearly 9 months, with regular outpatient follow‐up for a gradual reduction in prednisolone dosage. No complication has been related with ECMO use till this report.

Capture next‐generation sequencing (CNGS) was performed to detect the genetic basis. Genetic analysis revealed a mutation in the NLRP1 gene [c. (1175C>T) p. (Pro392Leu)] in heterozygosis, as reported in sJIA.^5^

## 3 DISCUSSION

The etiology of MAS in rheumatic disease remains unclear, but it is believed to be a complex genetic trait. MAS is characterized by a cytokine storm, which leads to dysregulated inflammatory activation of the immune system. Some MAS patients display atypical symptoms such as isolated neurological disease. Neurological symptoms are prominent at the onset of MAS and are mostly associated with abnormal CSF findings or an MRI scan of the brain. Without prompt diagnosis and appropriate treatment, it can rapidly progress to multi‐organ failure. In 2016, classification criteria for MAS in sJIA were introduced, providing a sensitive and specific tool for identifying MAS in this particular group of patients.^4^ It has been proposed that various inflammatory cytokines, including IL‐1, IL‐18, and IFN‐g in addition to IL‐6 play significant roles in the development of MAS. TCZ is widely used in the treatment of sJIA. After TCZ administration, serum IL‐6 levels typically increase significantly as TCZ inhibits IL‐6 receptor‐mediated consumption.^6^ However, early diagnosis of MAS still poses a clinical challenge. Traditional disease activity parameters such as CRP and serum ferritin are not sufficient to predict the development of MAS after TCZ therapy.

In the case we reported, the CRP and serum ferritin levels appeared to be within normal range prior to MAS. However, further investigation is needed to identify other disease parameters that can predict this occurrence. There have been reports suggesting that IL‐6 inhibition may be a contributing factor in altering the pathogenesis of MAS.^1^, ^2^ While we cannot definitively conclude that TCZ administration caused MAS in this patient, the findings from other cases as well as our own case suggest a potential association between TCZ treatment and the development of MAS in active sJIA patients.^1^

ECMO is increasingly utilized as a means to provide support for severe pulmonary and/or cardiac failure. The decision to employ ECMO in our case was based on the persistence of refractory hypoxia and a lack of significant improvement with mechanical ventilation.^7^ Our experience highlights the successful application of ECMO in managing MAS of sJIA patients presenting with severe respiratory failure unresponsive to standard ventilatory measures. To the best of our knowledge, this is the first report documenting successful remission in ECMO‐treated sJIA patients who developed acute pulmonary edema and refractory shock following TCZ infusion. Nonetheless, due to varying success rates, potential complications, and substantial associated costs, it is crucial to accurately identify critically ill patients who may be suitable candidates for the initiation of VA‐ECMO. A lot of complications have been reported with using of ECMO, including bleeding and thromboembolic events. In our case, there were no such complications.

TCZ‐induced anaphylaxis is not uncommon. Despite premedication with antihistamines and corticosteroids, the patient in our case still experienced anaphylaxis. The key approach in managing anaphylaxis to biologics is to avoid reusing the drug. Hence, patients with active disease characterized by high IL‐6 levels may be susceptible to developing anaphylaxis.

## 4 CONCLUSION

In conclusion, it is important to be vigilant for the occurrence of MAS and anaphylaxis when considering TCZ treatment, particularly in patients with sJIA. Intensive monitoring for these complications is crucial. Our report highlights the positive outcomes associated with ECMO in managing severe respiratory failure in sJIA patients.

## AUTHOR CONTRIBUTIONS

Xi Yang and Xuemei Tang wrote the manuscript. Xi Yang, Yingfu Chen, Rongxin Dai, Yunfei An, Xin Yan and Xue Tang managed the patients' care. Xi Yang, Xiaodong Zhao and Xuemei Tang revised the manuscript. All the authors read and approved the final manuscript.

## CONFLICT OF INTEREST STATEMENT

Prof. Xiaodong Zhao is the Deputy Editor‐in‐Chief of Pediatric Discovery. To minimize bias, he was excluded from all editorial decision‐making related tot the acceptance of this article for publication. The remaining authors declare no conflict of interests.

## ETHICS STATEMENT

An ethical application sent to the Ethics Committee of Chongqing Medical University was approved.

## CONSENT TO PARTICIPATE

Written informed consent was obtained from the parent of patient on January 29, 2022.

## CONSENT FOR PUBLICATION

Not applicable.

## Data Availability

The datasets generated during the current study are available from the corresponding author on reasonable request.
